# Genomic characterization and SNP analysis connect respiratory infections caused by Mycobacterium intracellulare with a pool facility

**DOI:** 10.1099/mgen.0.001577

**Published:** 2025-12-01

**Authors:** Caitlin A. Selway, Lisa Shephard, Michaela Hobby, Koen Vandelannoote, Chris Lease, David Cunliffe, Jamie Woodward, Timothy P. Stinear, Mark Turra, Simone Barry, James Geake, Richard Stapledon, Lito Papanicolas, Lex E. X. Leong

**Affiliations:** 1 Microbiology and Infectious Disease, SA Pathology, Adelaide, SA 5000, Australia; 2 Health and Regulation Protection, SA Health, Adelaide, SA 5000, Australia; 3 Department of Microbiology and Immunology, Doherty Institute, University of Melbourne, Melbourne, VIC 3000, Australia; 4 SA TB Services, Central Adelaide Health Network, Adelaide, SA 5000, Australia; 5 University of Adelaide, Adelaide, SA 5000, Australia; 6 Royal Adelaide Hospital, SA Health, Adelaide, SA 5000, Australia; 7 University of South Australia, Adelaide, SA 5000, Australia

**Keywords:** environmental opportunistic mycobacteria, *Mycobacterium intracellulare*, whole-genome sequencing

## Abstract

Non-tuberculous mycobacteria are emerging respiratory pathogens that can persist in treated water systems. In 2018, a cluster of *Mycobacterium intracellulare* lung infections was linked to a pool facility in Australia, prompting an epidemiological and genomic investigation. *M. intracellulare* was isolated from five sputum samples across four clinical cases and from fourteen pool water samples across a total of five collection time points. All cases were resolved following exclusion from the pool facility, with only one patient requiring short-term steroids; none of the patients required anti-mycobacterial treatment. To test if this was a point-source outbreak, whole-genome sequencing of mycobacteria recovered from patients and the pool was implemented. Initial analysis confirmed all patient and water isolates were *M. intracellulare* with sequence type 210. A complete, circular genome was constructed from one of the isolates linked to this cluster and was used as a reference genome for high-resolution core genome SNP analysis. This analysis showed tight clustering of *M. intracellulare* genomes from patient and pool water isolates that were distinct from other *M. intracellulare*. Thus, epidemiological and comparative genome analysis strongly implicated the pool as the origin of these infections.

## Data Summary

FASTQ files have been deposited to the National Centre for Biotechnology Information (NCBI) Sequence Read Archive. The complete genome sequence has been deposited in the NCBI Genome repository with BioSample ID SAMN44053086. All new samples published in this manuscript can be found under BioProject no. PRJNA1168590, with specific BioSample IDs listed in Table S1. The authors confirm all supporting data, code and protocols have been provided within the article or through supplementary data files.

**Impact Statement**

The non-tuberculous mycobacteria species, *Mycobacterium intracellulare*, is a respiratory pathogen that can persist in treated water systems. Through an epidemiological and genomic investigation, we report an outbreak of *M. intracellulare* lung infections in patients that traced back to a common point source – a pool facility used by all affected patients. Given the rising prevalence of pulmonary *Mycobacterium avium*–*intracellulare* complex disease, this investigation highlights the importance of clinical vigilance for patients presenting with respiratory infection symptoms, including careful clinical assessment, referring patients for mycobacterial sputum sampling and screening for exposure to communal water-based facilities. In conjunction, establishing routine and regular surveillance for mycobacterial pathogens in communal water facilities should be implemented to mitigate infection risks and safeguard public health.

## Introduction

Non-tuberculous mycobacteria (NTM) have become emerging pathogens that can cause both pulmonary infection and disease, including syndromes such as hypersensitivity pneumonitis. These pathogens usually inhabit a wide variety of environments, with water being the primary reservoir [1]. NTM have been persistently isolated from drinking water in Australia [2] despite multiple preventative measures, including filtration treatment and disinfection mainly through chlorination and chloramination but also ozone, UV irradiation and chlorine dioxide [3]. The persistence of these organisms occurs through the lipid-rich mycobacterial cell wall [4], by intracellular colonization of protozoa [5], and their ability to form biofilm [6].

*Mycobacterium intracellulare* is one of the species from the *Mycobacterium avium*–*intracellulare* complex (MAC). Members from the MAC are slow-growing acid-fast bacilli. Globally, *M. intracellulare* is one of the most common causes of NTM pulmonary disease [7,8], which typically manifests along a spectrum from bronchiolitis and progressive bronchiectasis to necrotizing cavitary pulmonary disease. Hypersensitivity pneumonitis driven by an immune-mediated response to mycobacterial antigens is also well described and may exist on this continuum [9]. While MAC infections and pulmonary disease can occur opportunistically in immune-suppressed patients, there have been multiple cases reported of *M. intracellulare*-associated pulmonary pathologies in immune-competent patients [9,11]. *M. intracellulare* and other MAC species have consistently been detected in the environment, and multiple studies have established the environment as a source of human infection by demonstrating identical genotypes in both clinical and environmental samples [2,12, 13].

The advent of next-generation sequencing has significantly enhanced the resolution for distinguishing between NTM sub-species and strains. Here, we report an *M. intracellulare* cluster characterized using whole-genome sequencing. All cases were traced back to a common point source – a pool facility used by all affected patients. We also sampled water from the pool facility to determine the relatedness of environmental isolates to clinical cases.

## Methods

### Demographics

We conducted a cluster investigation of culture-positive MAC isolates from four patients who had visited a pool facility in Australia. Age, gender, risk factors, sputa culture, radiological findings and clinical outcomes of the patients were collected. As part of the environmental investigation, water samples were collected through routine inspections at the pool facility at different time periods. During these inspections, ~500 ml of pool water (per site) was collected from multiple water outlet sites, and each sample underwent culturing to detect the presence of MAC.

### Microbiology

MAC was isolated from clinical sputum samples (*n*=5; patients=4) and environmental water samples (*n*=14) at the Mycobacterium Reference Laboratory of SA Pathology, South Australia, Australia. Environmental water samples of 500 ml were concentrated by centrifugation at 3,000 ***g*** for 15 min. Then environmental and clinical samples were processed using similar in-house mycobacterial culturing procedures, whereby samples were first decontaminated with 2% sodium hydroxide with sodium citrate and *N*-acetyl-l-cysteine and then neutralized with phosphoric acid. Decontaminated samples were inoculated on Löwenstein–Jensen media (Edwards, Narellan, NSW, Australia) and incubated at both 30 and 35 °C for 12 weeks. Samples were also inoculated in Mycobacterial Growth Indicator Tubes (MGIT), incubated manually at 30 and 35 °C for 6 weeks in the Bactec MGIT 960 automated broth-based system (Becton Dickinson, Cockeysville, Maryland 21030, USA). Positive cultures were confirmed as MAC using MALDI-TOF MS and further characterized using whole-genome sequencing.

### Whole-genome sequencing

DNA was extracted from sub-cultured MAC isolates on Löwenstein–Jensen media using the QIASymphony DSP Pathogen and Viral DNA kit. For short-read sequencing, genomic DNA was fragmented and amplified using Nextera XT library preparation (Illumina, Inc., California, USA), before being sequenced on a NextSeq 550 platform with a NextSeq 500/550 MID Output (2×150 cycles) kit.

To generate a reference genome for phylogenetic assessment, high molecular weight DNA was extracted from one clinical isolate using the MagAttract High Molecular Weight DNA Kit (QIAGEN, Hilden, Germany). The long-read library was prepared using the Native Barcoding Kit SQK-NBD114.96 (Oxford Nanopore Technologies, Oxford, UK) and sequenced on an Oxford Nanopore Technologies (ONT) PromethION 2 Solo platform with a PromethION R10.4.1 flow cell (FLO-PRO114M, Oxford Nanopore Technologies, Oxford, UK). The high molecular weight genomic DNA from the same clinical isolate was also sequenced on the Illumina platform as described above.

### Bioinformatic analysis

Sequencing quality was assessed using *seqtk* (v.1.3-R106) [14]. Species and sub-species identification was performed using high-quality Illumina short-read sequences using a kmer-taxonomic classification software, *Kraken2* (v2.1.2; database: k2_pluspf_20220607) [15], and an in-house generated mash (v2.3) [16] database containing sequences within the *M. intracellulare-chimaera* group. Multi-locus sequence typing (MLST) was performed using *mlst* (v2.19; mycobacteria scheme) [17] with the PubMLST database [18] available through https://pubmlst.org/.

Long-read sequences from ONT sequencing were base called with *dorado* (v0.6.2) [19] with the dna_r10.4.1_e8.2_400bps_sup@v4.3.0 model, followed by demultiplexing also using *dorado*. Using Illumina short reads and ONT long reads, a complete, closed genome was created using *hybracter* (v0.6.0) [20] via the hybrid approach. All parameters for the complete genome were set as default.

For core genome SNP analysis, short-read sequences from isolates in this investigation and other local *M. intracellulare* sequences from routine surveillance across 2016–2019, as well as contigs from MAC international representatives, were mapped to the generated closed genome, and SNP variants were called using *snippy* (v4.6.0) [21]. The core SNP multiple sequence alignment was generated using the *snippy-core* function of snippy with the auto-masking parameter to remove repetitive regions for three situations: (1) all sequences from the MAC international representatives, local *M. intracellulare* surveillance and the outbreak investigation; (2) local *M. intracellulare* surveillance and the outbreak investigation; and (3) only sequences from the outbreak investigation. The multi-sequence alignments were used to construct phylogenetic trees using *IQ-TREE* (v2.2.0) [22] with the following parameters: ModelFinder [23], 1,000 ultra-fast bootstraps [24] and fconst sites identified with *SNP-sites* [25]. The phylogenetic tree was visualized through ggtree (v3.6.2) [26]. Network analysis for the investigation subset was visualized through MicrobeTrace (https://microbetrace.cdc.gov/MicrobeTrace/). Pairwise SNP distances were determined using *snp-*dists (v0.8.2) [27], which were used for single-linkage clustering analysis with *R-stats* (v4.1.2) [28]. A broad cluster threshold of 50 SNPs was used to capture potential environmental variation over many months. Core genome SNP differences between clusters were identified and then annotated using *prokka* (v1.14.6) [29].

## Results

### Epidemiological and environmental analyses

Clinical samples (*n*=5) were collected from patients (*n*=4) between September and October 2018 and from environmental samples (*n*=14) between September 2018 and March 2019 (Table 1). Patients were aged between 43 and 85 and regularly attended the same pool facility. All patients presented with symptoms of respiratory infection, including cough, dyspnoea, fever and sweats. Contemporaneous medical imaging for patients 1, 2 and 4 demonstrated subtle changes characterized by small nodules and focal areas of ‘tree-in-bud’ change (Table 2).

**Table 1.** Clinical and environmental sample information from the pool facility *M. intracellulare* outbreak investigation

Sample ID	Date of collection	Sample type	Group	Sample description
48F1	13 September 2018	CLINICAL	Patient 1	Sputum
48F2	14 September 2018	CLINICAL	Patient 1	Sputum
90F1	12 October 2018	CLINICAL	Patient 2	Sputum
72M1	17 October 2018	CLINICAL	Patient 3	Sputum
64F1	26 October 2018	CLINICAL	Patient 4	Sputum
S0A1	28 September 2018	ENV	Collect 1	Water from leg to filter
S0B1	28 September 2018	ENV	Collect 1	Water from leg from filter
S0A2	28 September 2018	ENV	Collect 1	Water from leg to heater
S0C	28 September 2018	ENV	Collect 1	Water from middle of pool
S0B2	28 September 2018	ENV	Collect 1	Water from leg from the heater
S1B1	8 October 2018	ENV	Collect 2	Water from leg from filter
S1A2	8 October 2018	ENV	Collect 2	Water from leg to heater
S1C	8 October 2018	ENV	Collect 2	Water from middle of pool
S1B2	8 October 2018	ENV	Collect 2	Water from leg from the heater
S2A1	22 November 2018	ENV	Collect 3	Water from leg to filter
S3B1	9 January 2019	ENV	Collect 4	Water from leg from filter
S4A1	7 March 2019	ENV	Collect 5	Water from leg to filter
S4B2	7 March 2019	ENV	Collect 5	Water from leg from heater
S4B1	7 March 2019	ENV	Collect 5	Water from leg from filter

**Table 2.** Patient characteristics

Patient	Age	Gender	Baseline radiology/clinical disease	Positive sputum samples	Microscopy and culture
1	48	Female	Hypersensitivity pneumonitis	2 of 2	Smear positive; culture positive
2	90	Female	Hypersensitivity pneumonitis	3 of 3	Smear negative; culture positive
3	72	Male	Not available*	4 of 4	Smear negative; culture positive
4	64	Female	Hypersensitivity pneumonitis	1 of 1	Smear negative; culture positive

*Patient 3 did not have medical imaging available for review until December 2019.

**Fig. 1.**
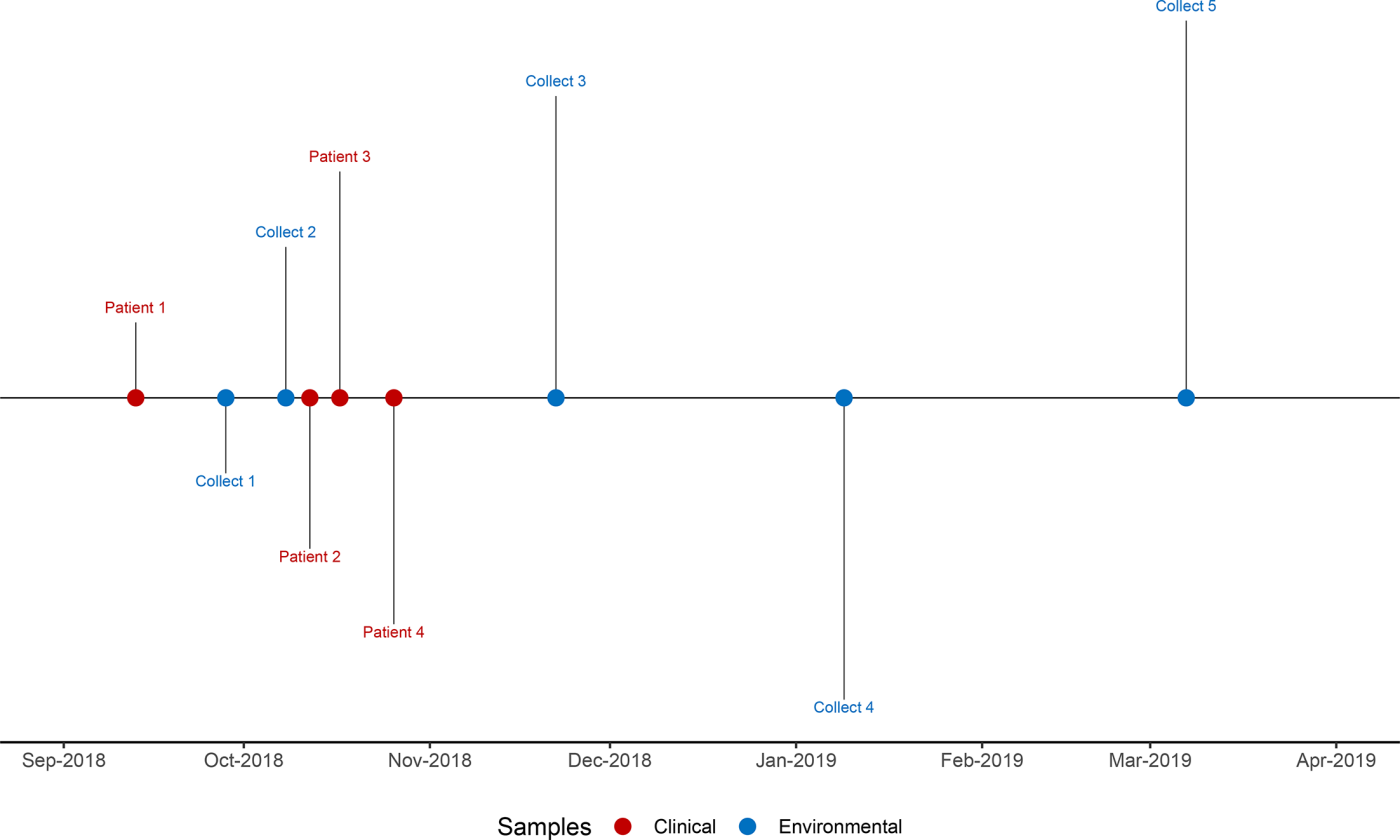
Epidemiology of the pool facility *M. intracellulare* outbreak investigation.

Sputum was collected from Patient 1 (48F1 and 48F2) on 13 September 2018, after the patient visited a general clinic with respiratory symptoms (Fig. 1). Following confirmation of MAC in sputum samples from Patient 1, water samples were collected from the pool facility by SA Health’s Health Protection and Regulation unit (Collect 1 and Collect 2; Table 1).

Briefly, ~1 month after the first case presentation, three more cases presented to a local tertiary hospital with similar symptoms (Patient 2: 90F1, Patient 3: 72M1 and Patient 4: 64F1). Water samples from the pool outlets were collected on three more occasions (Collect 3, 4 and 5; Table 1). All 14 water samples from the five collection visits and all five clinically cultured slow-growing mycobacteria were identified as MAC through MALDI-TOF.

Patient 1, who had the highest level of exposure, was referred to a respiratory physician and was assessed as most likely having hypersensitivity pneumonitis based on the clinical and high-resolution computed tomograph findings. This patient responded well after three weeks of exclusion from the pool facility and short-term use of oral corticosteroids. The remaining cases resolved clinically following exclusion from the pool facility, and no cases required anti-mycobacterial therapy, indicating that the illness was transient and consistent with a hypersensitivity or self-limited infection rather than progressive NTM pulmonary disease (NTM-PD).

### Genomic analysis and phylogenetic analysis of the mycobacterial isolation

The patient isolates and environmental isolates from the pool facility had on average 3,996,980 reads (range: 2,617,140–8,049,148 reads) per sample (see Table S1, available in the online Supplementary Material). All isolates were identified as *M. intracellulare* using both Kraken2 and the in-house mash database of *M. intracellulare-chimaera* group sequences, which confirmed that all isolates associated with the outbreak were *M. intracellulare*. All strains from this investigation had the same MLST, with sequence type 210.

To allow for more sensitive core SNP genome analysis, one clinical strain from the study was selected to produce a complete closed genome using both short- and long-read technologies. A total of 3,720,166 short-read Illumina sequences (total yield=556 Mb; average *Q*-score=31.4) and 294,708 long-read ONT sequences (total yield=803 Mb; average length=2,725 bp; average *Q*-score=29.0) were used to assemble the *M. intracellulare* genome. The assembled genome consisted of a closed, circular chromosome of 5,621,127 (98× coverage) with a G+C content of 67.89%. A total of 5,309 coding DNA sequences were identified within the genome. No plasmids were detected.

**Fig. 2.**
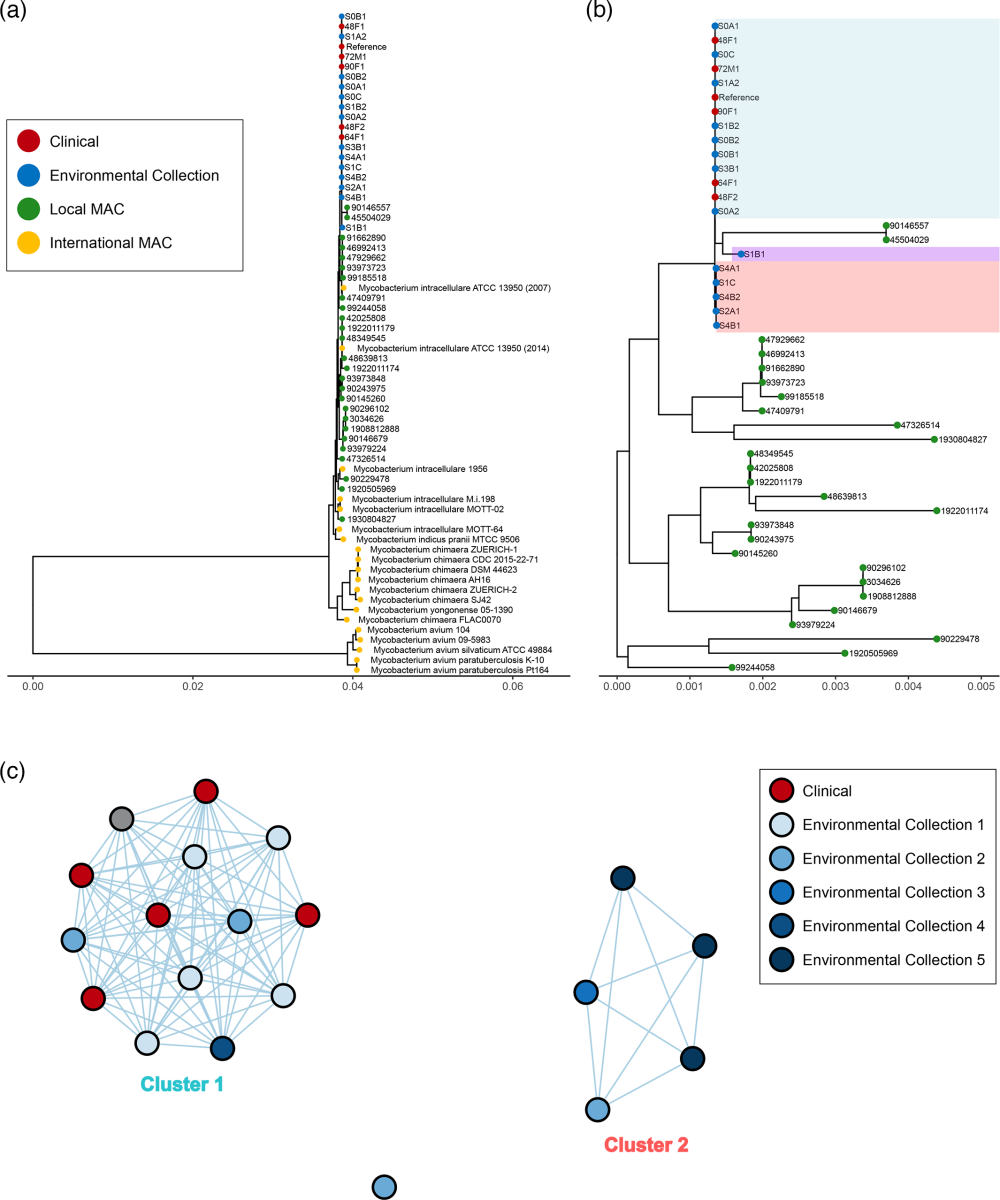
Genetic relatedness of the isolates from the investigation. Maximum likelihood phylogenetic trees constructed using the core SNP genome of *M. intracellulare* from this outbreak investigation along with (a) international MAC representatives and local *M. intracellulare* sequences collected between 2016 and 2019 (core SNP length=156,885 bp) and (b) local *M. intracellulare* sequences (core SNP length=79,667 bp). Clinical samples are represented by red circles, environmental samples by blue circles, local MAC samples by green circles and international MAC samples by yellow circles. Cluster 1 is coloured in light blue, and Cluster 2 is coloured in light red. A sample from Collect 2 with large SNP variation is coloured in purple. (**c**) A network analysis clustering clinical isolates with environmental isolates from this investigation (core SNP length=2,705 bp).

SNP analysis was used to further investigate the genetic relatedness of all isolates from this investigation, as well as in the context of a local dataset from 2016 to 2019 and globally (see Table S1 for sample details). Using the completed genome as the reference genome for SNP phylogenetic analysis, we identified that sequences from clinical and water isolates within the outbreak investigation form a monophyletic group with a minimum of 2,956 SNPs from global representatives (Fig. 2a) and 9,500 SNPs distance from other local *M. intracellulare* sequences (Fig. 2b).

To understand the genetic diversity of the outbreak, we performed SNP analysis with only the isolates from the investigation and determined a median pairwise distance of 0 SNPs (range=0–8) between the clinical isolates of *M. intracellulare* (Fig. 2c). In addition, all five isolates from water samples from Collect 1 and two isolates from Collect 2 were clustered with the clinical isolates at 0–3 SNPs (Cluster 1; Fig. 2c). In addition, one isolate from Collect 4 was closely related to isolates within Cluster 1 but was 16 SNPs from these isolates. Interestingly, one sample from Collect 2 and subsequent sample collections from Collect 3 and Collect 5 formed a separate cluster (Cluster 2; Fig. 2c) that was 310–370 SNPs from Cluster 1 and had a larger SNP range between the core genomes (cluster SNP median=3, range=0–48 SNPs). Further investigation showed no mutations in antibiotic or virulence factor genes for Cluster 2. One sample from Collect 2 had a much higher variation, with 2,316–2,678 SNPs distant from all other isolates associated with this outbreak.

### Discussion

In this cluster investigation, patients who presented with symptoms consistent with NTM-associated lung disease were found to have an *M. intracellulare* infection. All clinical cases acquired *M. intracellulare* from the same clonal expansion, with these isolates also being highly related to water samples from Collect 1 (Cluster 1). Overall, core SNP genome analysis confirmed the point source to be a pool facility frequently visited by all cases. While the direction of transmission could not be ascertained from this cluster investigation, *M. intracellulare* is not transmitted between humans [30]; therefore, the pool was the most likely source of infection.

MAC outbreaks and pulmonary infections from environmental exposure can occur, and prevalence estimates of pulmonary MAC disease have established a rising trend globally [31,34]. MAC has been routinely found in urban water distribution systems and household plumbing [35,37], which are likely due to the characteristics that MAC and other NTMs share. *M. intracellulare* and *M. avium* have been reported to adhere and form biofilms on a range of household plumbing materials, causing the colonization and persistence of these NTMs in urban water systems [6]. In addition, the thick mycolic cell wall allows these bacteria to survive chlorine exposure [4,38]. Nevertheless, susceptibility to chlorine could be influenced by growth stage, colony type and temperature [39].

This study demonstrated the persistence of *M. intracellulare* in the pool facility, despite collection of samples across four different time points. In addition, after the third collection of water samples, several mutations were observed within the dominant *M. intracellulare* subclone in the water systems (Cluster 2). No mutations were observed in antibiotic or virulence factor genes, suggesting that any cleaning processes used may not have caused antimicrobial resistance in the bacteria, and potential selective pressure may have been due to other environmental pressures.

One limitation of the cluster investigation is that the mode of transmission could not be definitively established. However, given that *M. intracellulare* is not transmitted between humans, and all patients responded to the removal from the pool environment (with a brief course of systemic glucocorticoids required in only one person), it is highly likely that the pool facility was a reservoir of *M. intracellulare*. Additionally, there may have been a selection bias in identifying cases as mild or asymptomatic infections that could have gone undetected.

This case highlights public health challenges in reducing the risk of exposure to or infection with NTM in community facilities, such as pools. Prevention of colonization and disinfection can both be very difficult. For example, cultures of *M. avium* require substantially higher concentration and time of exposure for disinfection doses with chlorine and ozone compared with *Escherichia coli*, and similar results are seen for other NTM [38,40]. Therefore, for patients presenting with symptoms of respiratory infection, it is important that healthcare professionals undertake careful clinical assessment, screening for exposure to communal water-based facilities. Further, they should retain a high index of clinical suspicion for NTM infection when considering diagnostic investigations, including referring patients for mycobacterial sputum sampling. This case also highlights the importance of healthcare jurisdictions retaining robust and centralized public health databases on NTM infections so that point sources of environmental infection can be identified and addressed appropriately.

Public water-based facilities, such as pools, should implement routine and regular surveillance for mycobacterial pathogens in collaboration with relevant public health units and accredited mycobacterial laboratories. Establishing standardized monitoring protocols, effective disinfection strategies and timely reporting mechanisms will be essential in mitigating infection risks and ensuring water safety.

Overall, this investigation highlights the importance of robust environmental monitoring and clinical vigilance for NTM in communal water facilities. Given the rising prevalence of pulmonary MAC disease, improved prevention and detection strategies are essential to reduce community exposure and safeguard public health.

## Supplementary material

10.1099/mgen.0.001577 Uncited Table S1.

## Abbreviations

MAC: *Mycobacterium avium*–*intracellulare* complex
MGIT: Mycobacterial Growth Indicator Tubes
MLST: multi-locus sequence typing
NTM: non-tuberculous mycobacteria
NTM-PD: Nontuberculous mycobacterial pulmonary disease
ONT: Oxford Nanopore Technologies
